# Cell fusion in the pathogenesis of COVID-19

**DOI:** 10.1186/s40779-021-00348-x

**Published:** 2021-12-20

**Authors:** You Zheng, Lu-Lin Zhou, Yan Su, Qiang Sun

**Affiliations:** Laboratory of Cell Engineering, Institute of Biotechnology; Research Unit of Cell Death Mechanism, Chinese Academy of Medical Science, 2021RU008, 20 Dongda Street, Beijing, 100071 China

## Dear Editor,

Infection with severe acute respiratory syndrome coronavirus 2 (SARS-CoV-2), which causes coronavirus disease 2019 (COVID-19), is initiated by the virus spike (S) glycoprotein binding to its cellular receptor such as angiotensin converting enzyme 2 (ACE2) [1, 2]. Subsequently, the virus may take two routes for its entry into the host cells, including an endocytic route that ends up in endosomal compartment, and a plasma membrane route on the cell surface. In both routes, fusion between the viral and plasma membrane was required to introduce the viral genome into the cytoplasm [3]. Similarly, SARS-CoV-2 infection results in the expression of fusogenic S glycoprotein on the host cell membrane, which upon binding to ACE2 on the neighboring cells, promotes cell–cell fusion to produce multinucleated syncytia [4, 5]. Syncytia were readily detected in the postmortem lung tissues of patients with COVID-19, but it remains unclear how syncytia might impact the pathogenesis of COVID-19, and whether syncytia formation might serve as a therapeutic target for COVID-19.

Recent studies have made great advances in the overall understanding of SARS-CoV-2 pathobiology, suggesting that syncytia are a definitive pathological characteristic of COVID-19 [2, 6]. In particular, by examining a set of postmortem lung tissues from 14 patients with COVID-19, Zhang et al. [2] found that the majority (10/14) of the samples had syncytia along with extensive damage in tissue structures. Moreover, most of the syncytia tended to internalize lymphocytes to form the unique cell-in-cell structures, followed by efficient death of the internalized lymphocytes as evidenced by time lapse imaging in cultured cells. The amounts of syncytia and cell-in-cell structures had a negative correlation with the number of lymphocytes in patient’s peripheral blood, indicating that syncytia might serve as a depleting unit for lymphocytes, conceivably contributing to the lymphopenia in patients with COVID-19. The idea was conceptually supported by an elegant in vitro co-culture experiment, where lymphocytes were co-cultured with control and syncytia-competent 293 T cells, respectively, for 24 h. The co-culture allows lymphocytes to be continuously internalized by syncytia, but not the control cells, to form heterotypic cell-in-cell structures, which led to constant consumption of lymphocytes. As a result, the number of lymphocytes progressively decreased in co-culture with syncytia over time, consistent with an active role of syncytia formation in lymphopenia of patients with COVID-19 [2]. Mechanistically, a bi-arginine motif (RxxR) in the S glycoprotein, which controls the production of the S2 fusion segment, was identified as a molecular switch that dictates the membrane fusion between cells. Interestingly, this bi-arginine motif is also present in the surface glycoproteins of other highly contagious viruses such as human immunodeficiency virus (HIV) and respiratory syncytial virus (RSV), but absent from those of less infectious viruses such as influenza virus A (H1N1) [2]. Consistently, syncytia were also observed during the infection of RSV and HIV, which took place along with lymphopenia as well. It is therefore anticipated that cell fusion induced by viral infection may be a shared mechanism promoting lymphopenia, and blocking the cleavage of surface glycoprotein may be a potential strategy to attenuate the pathogenic damage caused by the highly contagious viruses.

In addition to tissue damage and lymphopenia, syncytia formation during SARS-CoV-2 infection was also found to facilitate virus spreading. Zeng et al. [7] reported in a pre-print paper that high rate of cell–cell fusion mediated by the S glycoprotein contributed to the cell-to-cell transmission of virus. Similarly, some viruses, such as HIV, hepatitis C virus (HCV), and human cytomegalovirus (HCMV), promote cell fusion and the formation of multinucleated syncytia, leading to enhanced patient mortality. Compared with cell-free infection, cell-to-cell transmission was considered to be a more efficient way to escape host immune responses, particularly the antibody-mediated processes where the transmitted viruses were shielded from the extracellular antibodies. Thus, precise manipulation of the glycoprotein-induced cell–cell fusion may be critical to anti-viral therapies.

At present, a variety of SARS-CoV-2 inhibitors targeting viral entry are under intensive investigation. In the light of the pivotal roles of cell fusion in tissue damage, viral dissemination and immune evasion of COVID-19, it might serve as another potential therapeutic target. In fact, several candidate anti-SARS-CoV-2 compounds, such as hydroxychloroquine (HCQ), furin protease inhibitor (6-D-Arg), and cathepsin B/L inhibitor (E64D), were found to effectively block the processing of S glycoprotein in the production of fusogenic S2, and subsequent membrane fusion and lymphocyte internalization [2]. Interestingly, Braga et al. [6] found that TMEM16F, a chloride channel and lipid scramblase, was upregulated by S glycoprotein to induce membrane fusion. The inhibition of TMEM16F by chemical compounds such as Niclosamide could significantly impede S glycoprotein-inducing membrane fusion and syncytium formation [6]. Apart from chemical drugs, Xia et al. [8] reported a set of lipopeptides that also displayed high potency to inhibit pseudo-virus infection and membrane fusion mediated by SARS-CoV-2 S glycoprotein. Therefore, targeting cell fusion provides a novel avenue for the treatment and prevention of SARS-CoV-2 infection.

From the perspective of the rapid development of COVID-19 treatment strategies, additional work is clearly required, particularly with respect to the open question of whether combined administration of anti-syncytia drugs and other COVID-19 medicines might obtain better clinical effects. In this regard, the development of appropriate animal models would help elaborate the physiological functions of virus-induced syncytia in terms of viral transmission and dissemination, and escape from innate and adaptive immunity. Meanwhile, for other types of viruses, such as HIV, HCV, RSV and EBOV, it remains to be explored whether syncytia formation was associated with the viral pathology and infection persistence in their respective natural hosts.

## Data Availability

Not applicable.

## Abbreviations

ACE2: Angiotensin converting enzyme 2
COVID-19: Coronavirus disease 2019
EBOV: Ebola virus
HCQ: Hydroxychloroquine
HCMV: Human cytomegalovirus
HCV: Hepatitis C virus
HIV: Human immunodeficiency virus
RSV: Respiratory syncytial virus
SARS-CoV-2: Severe acute respiratory syndrome coronavirus 2
